# Development of a versatile TaqMan™ real-time quantitative PCR (RT-qPCR) compliant anchor sequence to quantify bacterial gene transcripts from RNA samples containing carryover genomic DNA

**DOI:** 10.1186/1472-6750-13-7

**Published:** 2013-01-31

**Authors:** Vijay J Gadkar, Martin Filion

**Affiliations:** 1 Department of Biology, Université de Moncton, 18 Antonine-Maillet, Moncton, NB, E1A 3E9, Canada

**Keywords:** Real-time quantitative PCR (RT-qPCR), DNase I, RNA, Anchor primed PCR, *Pseudomonas* spp, myIC, hcnC, phlD

## Abstract

**Background:**

In bacterial systems, the sequence congruence of genomic DNA (gDNA) and cDNA obtained following reverse transcription of RNA, makes gDNA an automatic target for qPCR primers. This could lead to aberrant gene expression quantification. This is why a rigorous treatment of bacterial RNA with DNase I is usually required to remove any traces of carryover gDNA. As bacterial RNA is known to be extremely labile, any procedure that affects RNA yield, such as DNase I treatment, can be logically assumed to also influence detection and quantification of gene transcripts, leading to either an underestimation or no detection at all. To address such problems, we have developed a novel and versatile TaqMan RT-qPCR compliant anchor sequence (MYT4) for quantifying bacterial gene transcripts without the need for DNase I treatment.

**Results:**

A non-genomic anchor sequence, henceforth referred to as MYT4 was designed using a synthetic DNA sequence called myIC, previously shown to share no significant homology to any known accession in the GenBank database. The sequence characteristic of MYT4 was kept within the design parameters required for the TaqMan RT-qPCR platform. The specificity and robustness of the novel MYT4 sequence was validated on RNA extracted from the bacterium *Pseudomonas* sp. LBUM300, grown under liquid culture and spiked soil conditions. Two transcripts, namely *hcnC* and *phlD*, were quantified from these two experimental systems. Using the MYT4 anchor, no RT-qPCR signal was detected from non-DNase I treated RNA, while strong signals were obtained using conventional reverse primers and RT-qPCR, indicating the presence of carryover gDNA in the RNA, extracted from either liquid culture or soil. Serial treatment of the RNA samples with DNase I (required to achieve absolute gDNA elimination) resulted in 50-70% loss of RNA which, when submitted to conventional RT-qPCR, significantly altered the transcript numbers detected when compared to the MYT4-based approach.

**Conclusions:**

Implementation of the versatile approach described in this study, which can be “retrofitted” to any existing TaqMan RT-qPCR system, should contribute to reducing the time and lowering the costs required to perform adequate bacterial RNA purification for downstream quantification of gene transcripts.

## Background

The real-time quantitative PCR (RT-qPCR) technique is now the most widely used analytical tool to detect and quantify gene expression in eukaryotic and prokaryotic organisms [1,2]. One unique feature of this technology is it’s exceedingly high (> 1000 fold) detection sensitivity *viz a viz* conventional agarose gel based detection [3-5]. This feature ironically also represents one of its major weakness since any carryover genomic DNA (gDNA) has the potential to contaminate the gene transcripts fluorescence signal, thereby skewing the final gene expression pattern [6,7].

Lack of introns in the prokaryotic genome makes it challenging to study bacterial gene expression using a highly sensitive technique like RT-qPCR [7,8]. In other words, the sequence congruence of the cDNA with its gDNA, makes the latter an automatic target for RT-qPCR primers, leading to an aberrant gene expression pattern. To alleviate such confounding effects of carryover gDNA, the main practical solution is to exhaustively treat the RNA sample with the enzyme DNase I [9-11]. This enzyme non-specifically cleaves the gDNA into 5′-phosphorylated di-, tri-, and oligonucleotide products [12], thereby making it an ineffective target for the RT-qPCR primers. Empirically, it is added to the RNA sample in pre-defined amounts, incubated for a period of *ca*. 15-30 min at 37°C, followed by a heat inactivation phase (65-70°C) for 5-10 minutes. Though usually effective, the whole work flow involved in this procedure, which includes handling of RNA at sub-optimal temperatures of 37°C and 65-70°C during incubation and denaturation, respectively, can lead to its hydrolysis, deleteriously affecting the overall integrity of the RNA preparation [13].

As repeatedly demonstrated earlier [14-17], multiple rounds of DNase I treatment is often required to make certain RNA samples amenable for RT-qPCR analysis. Such “higher-than-normal” gDNA load bearing samples are found in both pro- [14-17] and eukaryotic [18,19] systems. Empirical implementation of this multi-step approach is not only expensive, but also extremely laborious, especially under high-throughput conditions. Moreover, as RNA is not immediately converted to cDNA, the possibility of transcript loss remains high. For bacterial RNA’s, which are known to have an extremely short half life of few minutes [20], this delay can lead to RNA loss, leading to a possible underestimation of target transcripts.

One simple approach to reliably detect any transcript(s) using PCR from RNA samples containing gDNA is via an anchor priming strategy [21-23], whereby the reverse transcription primer is custom synthesized to contain an anchor sequence at its 5′ end. Reverse transcription using this modified primer results in the synthesis of the target cDNA’s having an anchor at its 5′ end. As a result, the target cDNA can then be specifically amplified by conventional end-point PCR using a target specific forward and an anchor specific reverse primer. In the present work, we have sought to apply this strategy to develop a versatile TaqMan RT-qPCR compliant anchor sequence for not only detecting but also quantifying microbial gene transcripts without the need for DNase I treatment. To assess the efficacy and demonstrate the superiority of this novel approach, spectrophotometric measurements and conventional TaqMan RT-qPCR were used in parallel to evaluate the loss of bacterial RNA during DNase I treatment and the downstream reduction in transcript numbers detected, respectively. To reduce/eliminate cross-reactivity issues of the novel anchor with gDNA targets, we used a synthetic DNA sequence which does not share any homology with any known sequences in the GenBank (NCBI) database [24,25]. The anchor sequence developed in this work was then tested on bacterial RNA, specifically on the expression of two biosynthetic genes, namely *hcnC* and *phlD*, from the well characterized bacterium *Pseudomonas* sp. LBUM300 [14,16,17,26]. To further evaluate its robustness and specificity, the developed anchor was also tested on RNA extracted from non-sterile agricultural soil spiked with the same bacterium.

## Methods

### Design of MYT4 anchor & specificity testing

The MYT4 anchor sequence was developed using the myIC [25] synthetic DNA construct [GenBank: FJ357008]. The design procedure was as follows: short stretches of the myIC sequence (*ca*. 20-25 bp in length) were selected at random and individually analyzed using the PrimerExpress v.3.0 software (Applied Biosystems, Foster City, CA). A specific utility of this software called the “primer/probe test” tool was used for this purpose. This tool interrogates any input sequence against a set of parameters defined for the TaqMan detection platform (ABI, Primer Express 3.0 user’s manual). Individual sequences that passed this test were identified and intensively analyzed using the BLASTn [27] search tool of the NBCI database. The candidate sequences that gave no or very low homology scores, especially against *Pseudomonas* spp. DNA sequences, were short listed. Of the six different candidate sequences identified, one sequence designated as “MYT4” (5′-CAGCTTGGTAGAATCGATCAGCTAC-3′) was chosen and used in the present study.

The specificity of the MYT4 anchor sequence was exhaustively tested on total RNA extracted from (a.) pure liquid culture of *Pseudomonas* sp. LBUM300 and (b.) non-sterile agricultural soil spiked with the same bacterium. The RNA was purposely not subjected to any DNase I treatment, so that an accurate estimation of the interaction of the MYT4 primer with the carryover gDNA could be assessed. PCR was performed using the same annealing temperature (60°C) as used for RT-qPCR (described below). The products of these PCR reactions were run on conventional 3% agarose gel electrophoresis.

### Bacterial culture experiment

Pure bacterial RNA was isolated from a liquid tryptic soy broth (TSB) culture of *Pseudomonas* sp. LBUM300, using the UltraClean Microbial RNA isolation kit (MoBio Laboratories, Carlsbad, CA, USA). The liquid culture was previously grown for 48 hours at 25°C with constant shaking at 250 rpm. Total RNA was quantified using the NanoDrop ND-1000 spectrophotometer (NanoDrop Technologies, Wilmington, DE).

### Soil spiking experiment

A previously characterized agricultural soil [14], sourced from the Agriculture and Agri-Food Canada’s S.H.J Michaud Research Farm (Bouctouche, NB, Canada) was used in the present study. The spiking procedure was essentially as described earlier [14], however only two- bacterial dilutions (1 × 10^9^ and 1 × 10^7^ bacteria/ml) and sampling points (0 and 7 days), were used. Briefly, a fixed amount of soil (20 g) was added to 50 ml tubes and inoculated with 4 ml of respective bacterial dilution or saline solution (non-spiked control). The tubes were manually shaken for 30 seconds. For each bacterial dilution ornon-spiked control, 8 replicate soil samples were prepared as described for a total of 24 samples and incubated in the dark at 25°C until sampling. At each sampling time point, 4 replicate samples per bacterial dilution and non-spiked control were used for bacterial RNA extraction (destructive sampling). Total RNA was extracted from 2 g of soil by using the Bürgmann’s method [28]. The final RNA pellet was dissolved in 100 μl of DEPC treated water and quantified by spectrophotometry (NanoDrop Technologies). To prevent any degradation during storage, the extracted RNA from either the liquid bacterial culture or soil, was used immediately for analysis.

### Multiple DNase I treatment of total RNA

The RNA extracted was either used directly for RT-qPCR analysis or, to achieve total elimination of carryover gDNA, each individual RNA sample was also subjected to three successive rounds of DNase I treatment as described earlier [16,17]. After each individual round, an aliquot of RNA was quantified by spectrophotometry (NanoDrop Technologies) for yield and purity and submitted to RT-qPCR analysis. The detailed workflow of this serial DNase I treatment of the test RNA sample is described in Additional file 1: Figure S1.

### Primer/probes design for RT-qPCR

**Figure 1:**
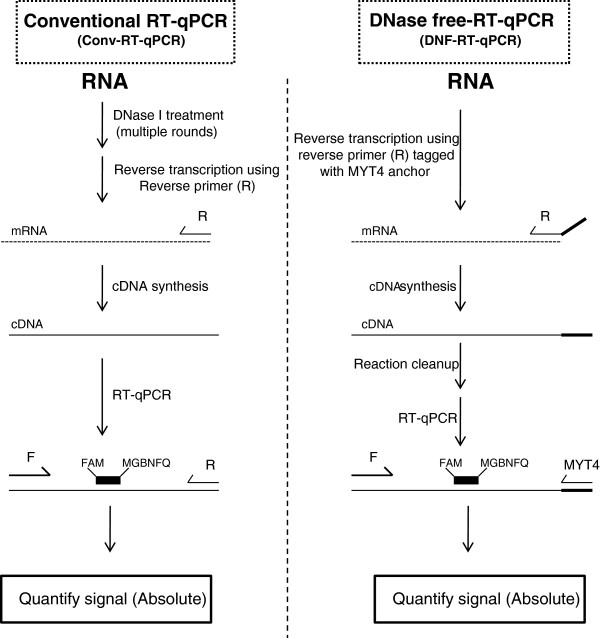
Schematic representation of conventional RT-qPCR (Conv-RT-qPCR) and DNase-free RT-qPCR (DNF-RT-qPCR).

For conventional RT-qPCR (Conv-RT-qPCR; Figure 1), the primers/TaqMan probes targeting the *hcnC* and *phlD* genes were as described earlier (14; Table 1). The *hcnC* and *phlD* genes are key biosynthetic genes involved in hydrogen cyanide and 2,4-diacetylphloroglucinol production, respectively, two compounds of interest for biological control of plant pathogens [14,17,26]. The same primers as those described for Conv-RT-PCR were used for the RT-qPCR analysis where the input RNA template was not subjected to DNase I treatment. This variant RT-qPCR is henceforth referred to as DNase-free-RT-qPCR (DNF-RT-qPCR; Figure 1). The only difference between DNF-RT-qPCR and Conv-RT-qPCR is the use of reverse primers tagged with the MYT4 anchor to introduce the MYT4 binding site (Figure 1). These reverse transcription specific primers were designated as PMYT4 and HMYT4 for the two target genes *phlD* and *hcnC*, respectively (Table 1).

**Table 1:** Primers and probes used in the present study

		**Target**	**Primer/probe**	**Sequence (5′ →3′)**	**Reference (GenBank Acc No.)**
conv-RT-qPCR		hcnC	hcnC-F	CCTGCCCCAGTCGTTCTTT	DeCoste et al (2011)(DQ788990)
			hcnC-R	TGCAACTGCGGATACATTGC	
			hcnC-FAM	FAM-ATTTCGCCTTGCAGTCC-MGBNFQ	
		phlD	phlD-F	CGGCGGACGGAAAATTC	DeCoste et al (2011)(DQ788986)
			phlD-R	CCGACCGGGTTCCAAGTC	
			phlD-FAM	FAM-TGATGAACTGGTCCTGCAA-MGBNFQ	
DNF-RT-qCPR		hcnC	hcnC-F	CCTGCCCCAGTCGTTCTTT	DeCoste et al (2011)(DQ788990)
			MYT4	CAGCTTGGTAGAATCGATCAGCTAC	
			hcnC-FAM	FAM-ATTTCGCCTTGCAGTCC-MGBNFQ	
			HMYT4^**¶**^	CAGCTTGGTAGAATCGATCAGCTACTGCAACTGCGGATACATTGC	Present work
		phlD	phlD-F	CGGCGGACGGAAAATTC	DeCoste et al (2011)(DQ788986)
			MYT4	CAGCTTGGTAGAATCGATCAGCTAC	
			phlD-FAM	FAM-TGATGAACTGGTCCTGCAA-MGBNFQ	
			PMYT4^**¶**^	CAGCTTGGTAGAATCGATCAGCTACCCGACCGGGTTCCAAGTC	Present work

^**¶**^: Reverse transcription primer. Underlined sequence is the binding site for the MYT4 primer.

MGBNFQ: Minor groove-binding nonfluorescent quencher.

### Production of standards for absolute quantification

**Figure 2:**
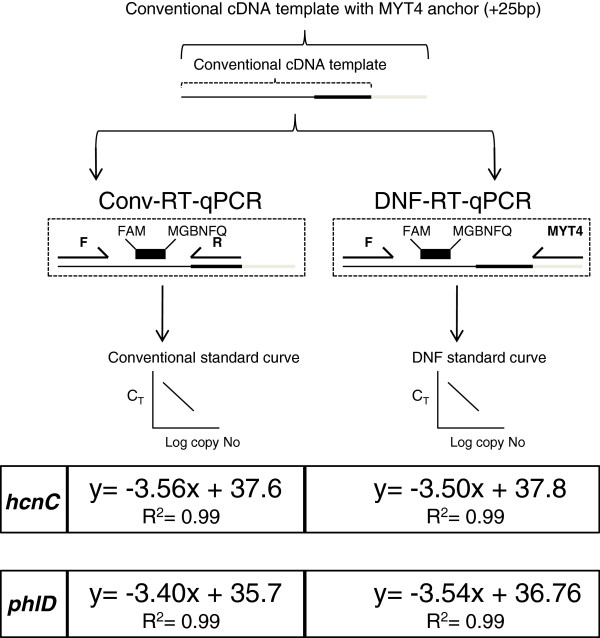
Amplification efficiency curves obtained in conventional RT-qPCR (Conv-RT-qPCR) and DNase-free RT-qPCR (DNF-RT-qPCR).

Since the cDNA generated using the HMYT4 and PMYT4 reverse primers also contain the binding site for the primers hcnC-R and phlD-R, respectively, a single standard for each gene was prepared (Figure 2). The standards for enumerating the absolute copy number of the targets were prepared by cloning the RT-qPCR amplicons obtained from of DNF-RT-qPCR based quantification (described in the earlier section) into the plasmid vector pKRX [29]. Plasmid copy number quantification and gene copy number calculation was done essentially as described earlier [14].

### cDNA synthesis & absolute quantification of *hcnC* and *phlD*

Reverse transcription reactions were performed by using the TaqMan Reverse Transcription kit (Applied Biosystems). Each RT reaction mix contained 7.9 μl of extracted RNA (200 ng), 2.0 μl of 10× RT Buffer, 4.4 μl (25 mM) of MgCl_2_, 4.0 μl (2.5 mM) of dNTPs, 0.8 μl (5 μM) of appropriate reverse primer, 8U of RNase inhibitor and 2.5U of Multiscribe RT enzyme for a final volume of 20 μl. For Conv-RT-qPCR, the hcnC-R and phlD-R primers were used for reverse transcription (Table 1), while PMYT4 and HMYT4 primers were used for DNF-RT-qPCR (Table 1). The cycling conditions used on a PTC200 Peltier ThermoCycler (MJ Research, Waltham, MA) were: 48°C for 30 min followed by 95°C for 5 min. Post-reverse transcription, reaction mixtures were cleaned using Qiagen PCR cleanup kit (Qiagen) and eluted in 20 μl of EB buffer.

Absolute quantification for both the target genes, namely *hcnC* and *phlD*, was performed using the TaqMan PCR Core Reagent Kit (ABI) on a MJ Research DNA Engine Opticon 2 (Bio-Rad, Mississauga, Canada). Each qPCR reaction mix (25 μl total volume) contained 6 μl of cDNA (1:5 dilution), 2.5 μl of 10× TaqMan Buffer, 5.5 μl (25 mM) of MgCl_2_, 0.5 μl (10 mM) of dATP, dCTP and dGTP, 0.5 μl (20 mM) of dUTP, 1.0 μl (5 μM) of probe, 2.5 μl (5 μM) of each primer, 0.25 μl AmpErase UNG (1 Unit/μl), 0.125 μl AmpliTaq Gold DNA Polymerase (5 Unit/μl) and 2.625 μl of DEPC-treated water (Ambion). Cycling conditions were: 50°C for 2 min; 95°C for 10 min; then 50 cycles of 95°C for 15 sec and 60°C for 1 min. Fluorescence was detected after each cycle.

### Data and statistical analysis

For absolute quantification, absolute transcript copy numbers for each gene were calculated using standard curves. For each gene and sampling date, the effect of bacterial concentration was analyzed by factorial ANOVA. For factorial ANOVA, *a posteriori* comparisons of the means between dilutions were done using Tukey-Kramer’s studentized range tests at a 5% level of significance. For pair-wise comparison, a Student’s t-test analysis was performed. All statistical analyses were performed using the CoStat software package ver. 6.20 (Cohort Software, Monterey, CA).

## Results

### Design & evaluation of the MYT4 anchor

The MYT4 anchor, designed on the synthetic myIC construct, gave very low homology to any known sequences in the GenBank database (“E” value parameter score ranging from 3 to 200). Specifically, no homology was found for the four terminal bases (3′ end) of the MYT4 anchor sequence with any known accessions in the GenBank database making it highly suitable as a PCR primer.

**Figure 3:**
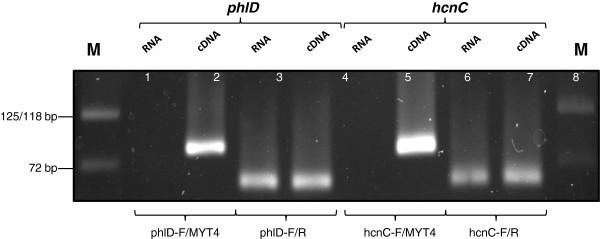
**PCR amplification of *hcnC *and *phlD *genes.** Agarose gel electrophoresis of *hcnC* and *phlD* genes, PCR amplified from RNA (non-DNase I treated) extracted from liquid culture of *Pseudomonas* sp. LBUM300. Primer system: phlD-F/MYT4 (Lane 1-2); phlD-F/phlD-R: (Lane 3-4); hcnC-F/MYT4 (Lane 5-6) and hcnC-F/R: Lane (7-8). Template: Non-reverse Transcribed RNA (Lane 1, 3, 5, 7); cDNA (Lane 2, 4, 6, 8). M: Quick-Load DNA marker, Broad Range (New England Biolabs, Mississauga, ON).

Empirical functioning of the MTY4 anchor was tested by conventional PCR on RNA extracted from *Pseudomonas* sp. LBUM300. When the phlD-F/R and hcnC-F/R primer pairs were used, positive amplicons were obtained not only from the cDNA, but also from the RNA (not DNase treated) templates (Figure 3). This strongly indicated the presence of carryover gDNA in the RNA preparation. When the MYT4 anchor primer was substituted for the reverse primers (phlD-R and hcnC-R), positive amplifications were observed only for the cDNA templates (Figure 3, Lane 2, 6). Similar results were obtained when RNA extracted from soil spiked with *Pseudomonas* sp. LBUM300 was tested using the same primer system (data not shown).

### Substitution of reverse primers with MYT4: effect on amplification efficiency

As the MYT4 primer is the only replacement during DNF-RT-qPCR, we evaluated if this replacement affected the overall efficiency of amplification (Figure 2). The amplification curves (Figure 2) obtained using this primer replacement was highly similar for both *phlD* and *hcnC*. As a result, it was possible to use a single standard for each gene during both Conv-RT-qPCR and DNF-RT-qPCR.

### Impact of multiple DNase I treatment on RNA yield

**Figure 4:**
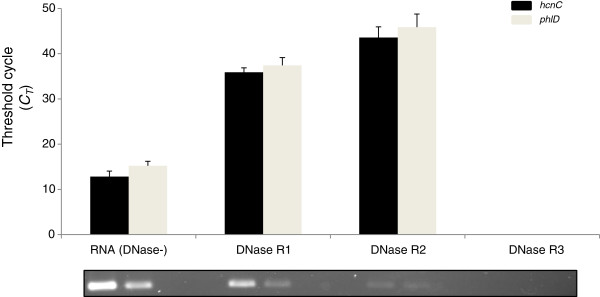
**Conventional qRT-PCR assays of *hcnC *and *phlD*genes using non-treated (RT minus control assay) and DNase-treated RNA samples.** DNase R1, R2 and R3 represent first, second and third round of DNase treatment of RNA extracted from liquid bacterial culture of *Pseudomonas* sp. LBUM300. Bars are standard error of the mean. PhlD-F/R and hcnC-F/R primer combination used for amplification of *phlD* and *hcnC* genes, respectively. An aliquot of the RT-qPCR reaction was run on 3% agarose gel to verify the amplification (bottom panel).

Purity of total RNA extracted from both liquid bacterial culture and soil spiked with *Pseudomonas* sp. LBUM300 was confirmed by spectrophotometric readings. The average *A*_*260*_/*A*_*280*_ values for RNA extracted from liquid bacterial culture and soil was 1.88 and 1.72, respectively. Direct submission of the RNA (minus DNase treatment) from both the matrices to Conv-RT-qPCR analysis (RT minus assay) gave *C*_*T*_ values in the 10-15 cycle range (Figure 4). This strongly indicated the presence of carryover gDNA. When the same RNA samples were subjected to serial DNase I treatment, the *C*_*T*_ values changed to 35-38 (DNase R1), 41-42 (DNase R2) and no detection (DNase R3), when analyzed under similar assay conditions, i.e. RT minus assay using Conv-RT-qPCR (Figure 4). Aliquots of the RT-qPCR mixture when resolved on a 3% agarose gel electrophoresis showed positive amplicon formation of correct size (Figure 4). The intensity of these amplicons was diminished with serial DNase I treatment. No signal whatsoever was detected when the same RNA samples were analyzed by DNF-RT-qPCR (i.e. the reverse primer hcnC-R or phlD-R was substituted with MYT4). Similar results were obtained for RNA extracted from soil spiked with the test bacterium (data not shown).

For RNA extracted from a liquid bacterial culture of *Pseudomonas* sp. LBUM300, the amount of RNA lost, after three rounds of serial DNase I treatment, was *ca*. 79.0% (Table 2). Similarly, the RNA extracted from natural soil, both spiked/non-spiked with the test bacterium, when subjected to multiple rounds of DNase I treatment, also showed a loss of RNA after each round (Table 3). On average, there was a 35% and 49% loss of RNA for soil samples spiked with 1 × 10^7^ bacteria/ml at Day 0 and Day 7 respectively (Table 3). Similarly, the RNA loss was *ca*. 60% in soils spiked with 1 × 10^9^ bacteria/ml at both Day 0 and Day 7 sampling points (Table 3). RNA in the control (non-spiked) samples was also lost at both the sampling dates and this loss was in the 50-55% range (Table 3).

**Table 2:** **Quantification of total RNA extracted from a 48 h old liquid culture of *Pseudomonas *sp. LBUM300 after each round of DNase I treatment**

**DNase I treatment**	RNA (ng/μL)*	**Percentage of RNA recovered after each round**^**¶**^
Round 0	247.2 ± 1.3	-
Round 1	130.3 ± 0.55	−47.3%
Round 2	84.2 ± 1.77	−65.9%
Round 3	52.3 ± 2.38	−78.8%

*: Avg of four replicates ± standard error of mean.

^**¶**^: values in % with respect to original starting amount after each round of DNase-I treatment.

**Table 3:** **Quantification of RNA extracted from soil spiked with two concentrations of *Pseudomonas *sp. LBUM300 after multiple rounds of DNase I treatment**

**Days post inoculation**	**Bacterial spiking amount**	RNA amount post DNase-I treatment (ng/μL)^*¶^			
		**Round 0**	**Round 1**	**Round 2**	**Round 3**
Day 0	Control	33.7 ± 1.7	28.2 ± 2.4	25.2 ± 1.7	16.5 ± 2.2
			(-16.3%)	(-25.2%)	(-51.0%)
	10^7^ bacteria/mL	53.9 ± 2.4	45.3 ± 3.1	40.1 ± 3.8	34.7 ± 4.2
			(-15.9%)	(-25.6%)	(-35.6%)
	10^9^ bacteria/mL	102.4 ± 2.9	82.2 ± 4.4	61.7 ± 3.3	40.7 ± 4.9
			(-19.7%)	(-39.7%)	(-60.2%)
Day 7	Control	34.4 ± 2.3	30.7 ± 2.8	26.8 ± 3.8	22.2 ± 4.5
			(-10.7%)	(-22.1%)	(-54.9%)
	10^7^ bacteria/mL	56.6 ± 3.2	43.8 ± 4.4	32.7 ± 4.6	29.1 ± 3.5
			(-22.6%)	(-42.2%)	(-48.5%)
	10^9^ bacteria/mL	59.5 ± 2.5	42.7 ± 3.2	34.9 ± 4.7	23.1 ± 5.4
			(-28.2%)	(-41.3%)	(-61.1%)

* : Avg of 3 replicate samples ± standard error of mean.

^**¶**^: values in brackets indicate % reduction in RNA amounts (with respect to original starting amount), recovered after each round of DNase-I treatment.

### Absolute quantification of *phlD* and *hcnC* gene expression

For each gene, the transcripts were quantified into absolute copy numbers, normalized to the amount of starting substrate, i.e. per μL for liquid bacterial culture and per g for the soil spiking experiment. In both the experimental setups, despite significant loss of RNA (after three rounds of DNase I treatment), the target transcripts could be detected using both RT-qPCR systems. The number of rounds of DNase I treatment that was applied had a significant impact on the final transcript number.

**Figure 5:**
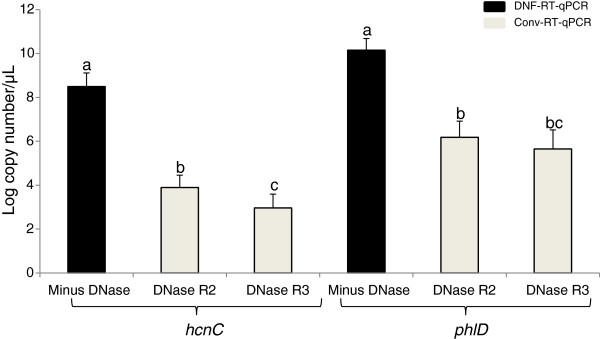
**Absolute quantification of *phlD *and *hcnC *gene transcripts from liquid bacterial culture of *Pseudomonas *sp. LBUM300.** Bars are standard error of the mean. X-axis indicates the no. of rounds of DNase I treatment performed on the RNA sample. Values followed by a different letter are significantly different using Tukey-Kramer test (P ≤ 0.05).

**Figure 6:**
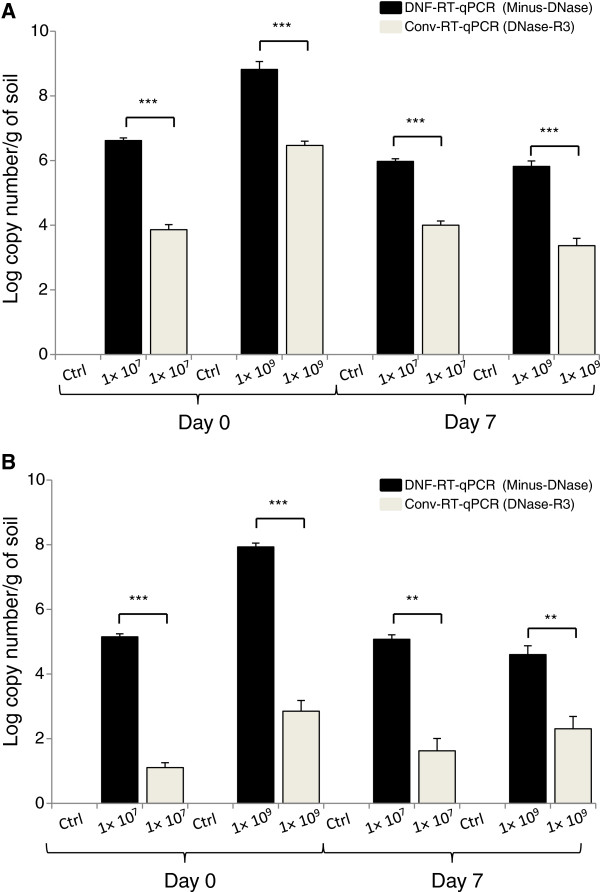
**Absolute quantification of (A) *hcnC *and (B) *phlD *gene transcripts in *Pseudomonas *sp. LBUM300 under soil conditions.** The *hcnC* gene transcripts expressed as copy number/g of soil at day 0 and day 7 post-inoculation. Bars are standard error of the mean. X-axis indicates the amount of bacterial cells inoculated at day 0 of the experiment. Results of pair-wise Student’s t-test. Significant differences are indicated by P ≤ 0.001 = ***.

In the non-DNase I treated RNA (“Minus DNase”) extracted from liquid bacterial culture, the *hcnC* and *phlD* transcript numbers (copies/μL) quantified using DNF-RT-qPCR were 3.10 × 10^8^ and 1.38 × 10^10^ copies/μL, respectively (Figure 5). These numbers were significantly lower (P ≤ 0.05) when the same transcripts were detected by Conv-RT-qPCR after two (DNase R2) and three (DNase R3) rounds of DNase I treatment (Figure 5). RNA samples not submitted to or submitted to only one round of DNase I treatment were not used for Conv-RT-qPCR analysis as they contained unacceptable levels of carryover gDNA (*C*_*T*_ ≤ 40; Figure 4). In the soil spiking experiment, the sampling date and number of rounds of DNase I treatment played an important role in determining the level of target transcripts detected. The *hcnC* (Figure 6A) and *phlD* (Figure 6B) gene transcripts were detected at both sampling time points, i.e. day 0 and day 7. Higher copy number of *hcnC* (Figure 6A) and *phlD* (Figure 6B) gene transcripts were consistently detected by DNF-RT-qPCR than Conv-RT-qPCR. This higher detection by the DNF-RT-qPCR system was regardless of the initial bacterial concentration or sampling date. A student’s t-test analysis between the copy number of transcripts detected by each of the RT-qPCR system showed a statistical difference, which ranged from very (p ≤ 0.01) to highly (p ≤ 0.001) significant (Figure 6A, 6B). Regardless of the RT-qPCR system used, no positive amplification was obtained on total RNA extracted from non-spiked soils.

## Discussion

In this study, we successfully developed a versatile TaqMan RT-qPCR compliant anchor sequence (MYT4) for quantifying microbial gene transcripts without the need for DNase I treatment. The specificity and robustness of the novel MYT4 sequence was validated on RNA extracted from the bacterium *Pseudomonas* sp. LBUM300 grown under liquid culture and spiked soil conditions, using a novel DNA free RT-qPCR approach (DNF-RT-qPCR). The superiority of the DNF-RT-qPCR approach was demonstrated using spectrophotometric measurements and conventional TaqMan RT-qPCR (Conv-RT-qPCR) used in parallel to evaluate the loss of bacterial RNA during conventional DNase I treatment and the downstream reduction in transcript numbers detected.

As expected, RNA extracted from both the liquid bacterial culture and soil was found to contain varying levels of carryover gDNA, as seen by the positive amplicons (Figure 3) and low *C*_*T*_ values (Figure 4) obtained from “RT minus” samples. To make these RNA samples amenable for conventional RT-qPCR analysis, DNase I treatment was clearly required. There are however no specific guidelines, at least to the best of our knowledge, which define that a test RNA sample should possess a certain *C*_*T*_ cut-off so that it becomes acceptable for RT-qPCR analysis. The only guidelines which pertain to RT-qPCR analysis are the MIQE [30,31] which sets certain benchmarks on the integrity of the input RNA template and not the status of the carryover gDNA, i.e. *C*_*T*_ cut-off value. Due to the lack of any specific parameters, we in this study decided to investigate the effect of a rigorous gDNA elimination step, i.e. DNase I treatment, on the final transcript numbers detected. To achieve this, we took an “absolute” gDNA elimination approach, wherein the test RNA sample was made totally free of carryover gDNA by multiple rounds of DNase I treatment (Additional file 1: Figure S1). Empirically, the efficacy of each DNase I treatment was tested by using the Conv-RT-qPCR approach on the RNA template (“RT minus control”).

The number of rounds of DNase I treatment required for an absolute gDNA elimination was highly dependent on the type of extraction matrix. While two rounds were sufficient for RNA extracted from liquid bacterial culture, as also observed previously [14,16], three rounds were required for natural soil for absolute gDNA elimination. This elimination was however at the cost of RNA yield which declined after each round of DNase I treatment (Tables 1 and 2). A single round of DNase I treatment of RNA extracted from liquid bacterial culture resulted in *ca* 47.0% loss of RNA, which increased considerably when the same RNA sample was subjected to a second (–65.9% loss) and a third (–78.8% loss) round of DNase I treatment (Table 2). Similarly, loss of RNA was observed when total RNA extracted from soil spiked with the test bacterium *Pseudomonas* sp. LBUM300 was subjected to multiple rounds of DNase I treatment (Table 3). To rule out RNA hydrolysis due to heat/cation [13] or organic based procedure (phenol/proteinase K), two of the commonly used procedures to inactivate the DNase I enzyme, we in our study made use of a commonly used proprietary, non-thermal/cationic inactivation system (http://tools.invitrogen.com/content/sfs/manuals/cms_055740.pdf). Despite this, there was an appreciable amount of RNA loss and the best explanation for this could be the high turnover rate amongst bacterial RNA’s and presence of the ubiquitous RNases in the sample. To develop a quantitative understanding of this loss and its effect on the transcript numbers detected, we implemented a novel highly sensitive DNF-RT-qPCR system. However, presence of carryover gDNA makes it difficult to quantify the target transcript from RNA samples which have not been subjected to any DNase I treatment. To circumvent the aforementioned limitation, we in the present work made use of an anchor priming strategy which allows PCR detection of transcripts in presence of any carryover gDNA [21-23].

The first step towards implementing the anchor priming approach was the identification of an anchor sequence, suitable for the TaqMan RT-qPCR platform. Direct adaptation of the earlier published anchors [21,23,32-34] was either not possible because of the proprietary nature [35] of the sequences or their lack of conformity to the strict primer design guidelines set for the TaqMan RT-qPCR platform. To address these two factors, we chose the 200 bp myIC sequence, a previously developed synthetic DNA construct as the starting point of our anchor design [24,25]. The myIC sequence does not share homology with any known accession in the GenBank database, making it an ideal candidate for experimental systems which demand an absolute negligible/no cross-reactivity. Interestingly, addition of the MYT4 anchor to the existing reverse primer did not alter the efficiency (Figure 2) *viz a viz* conventional primers, i.e. phlD-F/R and hcnC-F/R. This feature makes it very attractive to “retrofit” to any existing reverse primers, designed for the TaqMan RT-qPCR platform. Considering the similar range of conditions (melting temperatures, cycle number, etc.) generally used in SYBR Green-based RT-qPCR assays, we can speculate that the MYT4 anchor described in the present work could also most probably be used in SYBR Green-based amplification systems.

Regardless of the matrix used, three rounds of DNase I treatment of the RNA resulted in no fluorescence detection of any carryover gDNA when analyzed using the phlD-F/R and hcnC-F/R primer system (Figure 4). However in quantitative terms, this triple treatment had a significant negative impact on the final transcript numbers. For e.g. the *phlD* and *hcnC* gene transcripts (copy number/μl) in triple DNase I treated RNA samples extracted from pure bacterial culture were found to be 4.46 × 10^5^ and 9.12 × 10^2^, respectively (Figure 5). These amounts were substantially less then when the same RNA samples not subjected to any DNase I treatment were analyzed using the DNF-RT-qPCR system (1.38 × 10^10^ for *phlD* and 3.10 × 10^8^ for *hcnC*) (Figure 5). In other words, multiple or even a single rounds of DNase I treatment not only results in the loss of bacterial RNA, but substantially lowers one’s ability to sensitively detect a particular target transcript. A similar trend was observed from RNA samples extracted from spiked soil when subjected to multiple rounds of DNase I treatment (Figures 6A and 6B).

## Conclusion

In summary, this work contributes to providing a better understanding of RNA loss occurring during the unavoidable gDNA elimination phase (DNase I treatment), especially when working with highly labile bacterial RNA. As observed in the present work, RNA losses could reach 50-70% post-DNase I treatment, significantly impacting on the RT-qPCR gene transcript quantification results. This not only alters the biological interpretation of the results obtained but also increases the chances of obtaining negative quantification results for samples with low transcript levels. To avoid such pitfalls, we suggest, at least during the initial validation phase, a rigorous implementation of the MYT4 anchor. This would allow researchers to take an informed decision on the validity of their Conv-RT-qPCR data or highlight the necessity of using the DNF-RT-qPCR system developed in this study for their final analyses. To make this approach more “main stream”, we further propose the development of benchmark guidelines similar to the MIQE [31] and MIAME [36] for testing the elimination of gDNA in RNA samples. In light of the results obtained in this study, elimination of gDNA co-eluted during the RNA extraction process should be properly evaluated in every system under study and proper controls included, which show complete elimination of gDNA prior to downstream analyses of gene transcripts, especially if Conv-RT-qPCR is to be used. Alternatively, implementation of the DNF-RT-qPCR approach described in this study could contribute to reducing the time and lowering the costs required to perform adequate bacterial RNA purification, eliminating the DNase I treatment step usually required post-RNA extraction.

## Abbrevations

PCR: Polymerase chain reaction; qPCR: Quantitative real-time PCR; RT: Reverse transcription; gDNA: Genomic DNA; DNF-RT-qPCR: DNase-free RT-qPCR; Conv-RT-qPCR: Conventional RT-qPCR.

## Competing interests

The authors declare that they have no competing interests.

## Authors’ contributions

VJG did the conceptual development, including the experimental aspects of the DNF-RT-qPCR, and wrote the manuscript; MF coordinated the study, including its analysis, and edited the manuscript. Both the authors read and approved the final manuscript.

## Supplementary Material

Additional file 1: Figure S1 Workflow for multiple rounds of DNase I treatment. Click here for file
