# Supplementary figures and images for: Development of a versatile TaqMan™ real-time quantitative PCR (RT-qPCR) compliant anchor sequence to quantify bacterial gene transcripts from RNA samples containing carryover genomic DNA
Source: BMC Biotechnol. 2013 Jan 31;13:7. doi: 10.1186/1472-6750-13-7 (PMC3689636; doi:10.1186/1472-6750-13-7)

# Workflow for multiple rounds of DNase I treatment

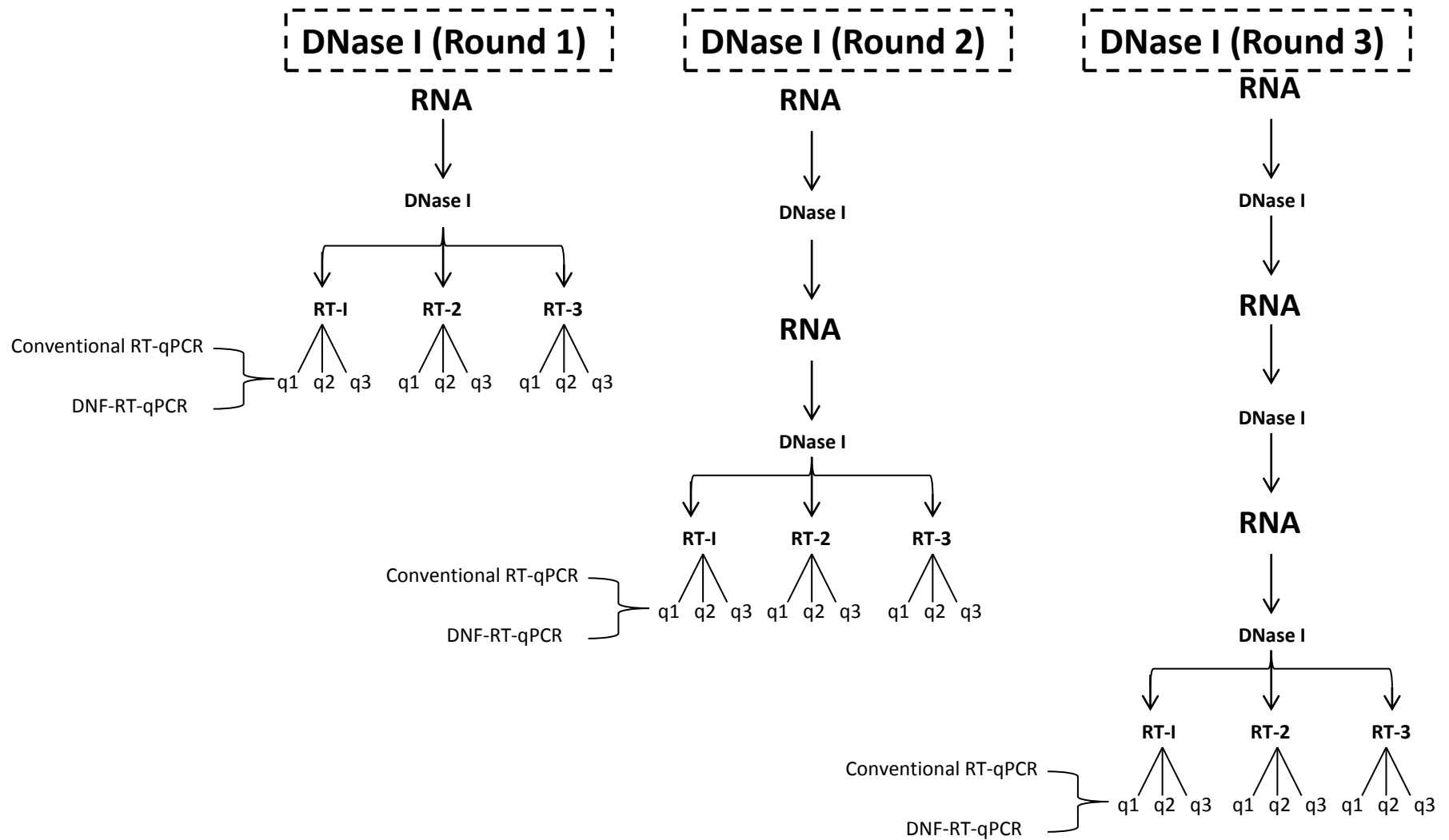

**Fig. 1\_Supplementary**

Supplement: Additional file 1: Figure S1 — Workflow for multiple rounds of DNase I treatment. [file 1472-6750-13-7-S1.pdf]
